# NF-*κ*B pathway link with ER stress-induced autophagy and apoptosis in cervical tumor cells

**DOI:** 10.1038/cddiscovery.2017.59

**Published:** 2017-09-11

**Authors:** Xiaolan Zhu, Li Huang, Jie Gong, Chun Shi, Zhiming Wang, Bingkun Ye, Aiguo Xuan, Xiaosong He, Dahong Long, Xiao Zhu, Ningfang Ma, Shuilong Leng

**Affiliations:** 1 Department of Human Anatomy, School of Basic Medical Sciences, Guangzhou Medical University, Guangzhou, Guangdong 511436, People’s Republic of China; 2 Guangdong Province Key Laboratory of Medical Molecular Diagnosis, Guangdong Medical College, Zhanjiang/Dongguan, People’s Republic of China; 3 Key Laboratory of Protein Modification and Degradation, School of Basic Medical Sciences, Affiliated Cancer Hospital and Institute of Guangzhou Medical University, Guangzhou 511436, People’s Republic of China

## Abstract

Targeting endoplasmic reticulum (ER) stress is being investigated for its anticancer effect in various cancers, including cervical cancer. However, the molecular pathways whereby ER stress mediates cell death remain to be fully elucidated. In this study, we confirmed that ER stress triggered by compounds such as brefeldin A (BFA), tunicamycin (TM), and thapsigargin (TG) leads to the induction of the unfolded protein response (UPR) in cervical cancer cell lines, which is characterized by elevated levels of inositol-requiring kinase 1*α*, glucose-regulated protein-78, and C/EBP homologous protein, and swelling of the ER observed by transmission electron microscope (TEM). We found that BFA significantly increased autophagy in tumor cells and induced TC-1 tumor cell death in a dose-dependent manner. BFA increased punctate staining of LC3 and the number of autophagosomes observed by TEM in TC-1 and HeLa cells. The autophagic flux was also assessed. Bafilomycin, which blocked degradation of LC3 in lysosomes, caused both LC3I and LC3II accumulation. BFA initiated apoptosis of TC-1 tumor cells through activation of the caspase-12/caspase-3 pathway. At the same time, BFA enhanced the phosphorylation of I*κ*B*α* protein and translocation into the nucleus of NF-*κ*B p65. Quinazolinediamine, an NF-*κ*B inhibitor, attenuated both autophagy and apoptosis induced by BFA; meanwhile, it partly enhances survival of cervical cancer cells following BFA treatment. In conclusion, our results indicate that the cross-talk between ER stress, autophagy, apoptosis, and the NF-*κ*B pathways controls the fate of cervical cancer cells. Careful evaluation should be given to the addition of an NF-*κ*B pathway inhibitor to treat cervical cancer in combination with drugs that induce ER stress-mediated cell death.

## Introduction

Endoplasmic reticulum (ER) stress is associated with the progression of cancer. Cellular adaptation to ER stress is mediated by the unfolded protein response (UPR).^1,2^ The UPR is mainly induced by three signaling sensors, inositol requiring enzyme 1*α* (IRE1*α*), protein kinase R-like ER kinase (PERK), and activating transcription factor 6*α*. These UPR signaling sensors are negatively regulated by the chaperone glucose-regulated protein-78 (GRP78)/BIP in the unstressed state. ER stress causes Grp78/BIP to release the sensors, thereby eliciting UPR.^3^ The function of the UPR is to re-establish ER homeostasis by regulation of components of the ER folding machinery and protein quality. However, when ER stress is unbearable or cannot be resolved, the UPR turns from a prosurvival to a prodeath response.^4,5^ Unfortunately, the molecular details of life/death decisions during ER stress are still too limited, and pathways whereby ER stress promotes cell death remain to be fully elucidated.

Similar to the UPR, macroautophagy (hitherto referred to as ‘autophagy’) is an adaptive response in tumor cells under environmental stress. Autophagosomes are double-membrane vesicles that mediate the first step of autophagy by sequestering damaged organelles and long-lived proteins. Autophagosomes mature by fusing with lysosomes (thereby becoming the so-called ‘autolysosomes’), which leads to the degradation of their contents.^6,7^ Autophagy has a close relationship with the programmed cell death pathway; further, uncontrolled autophagy itself can directly induce cell death through a process termed autophagic cell death.^8,9^

The human papillomavirus (HPV) is considered to be the major cause of cervical cancer,^10^ yet viral infection alone is not sufficient for cancer progression. Activation of the NF-*κ*B signaling pathway promotes proliferation, invasion and metastasis of cervical cancer cells. Thus, NF-*κ*B pathway inhibitors are being considered as potential anticancer agents in cervical carcinoma.^11–14^ UPR signaling sensors provide a potential link between the activation of the NF-*κ*B pathway, which regulates the expression of various proinflammatory genes and immunomodulatory molecules, and ER stress.^15^

For these studies, we postulated that inhibition of NF-*κ*B activation may represent a potential and safe target in the development of novel agents to treat cervical carcinoma cells. To test this hypothesis, we induced ER stress in cervical tumor cells using brefeldin A (BFA), tunicamycin (TM), or thapsigargin (TG), to trigger ER stress-mediated cell death. We found that ER stress significantly increased the UPR and led to the death of cancer cells by concomitant induction of autophagy in TC-1 tumor cells and HeLa cells by activating the NF-*κ*B pathway. Quinazolinediamine (QNZ), an NF-*κ*B inhibitor, decreased the autophagy and apoptosis induced by BFA.

## Results

### ER stress inducer (BFA, TM, and TG) lead the UPR in cervical tumor cells

**Figure 1:**
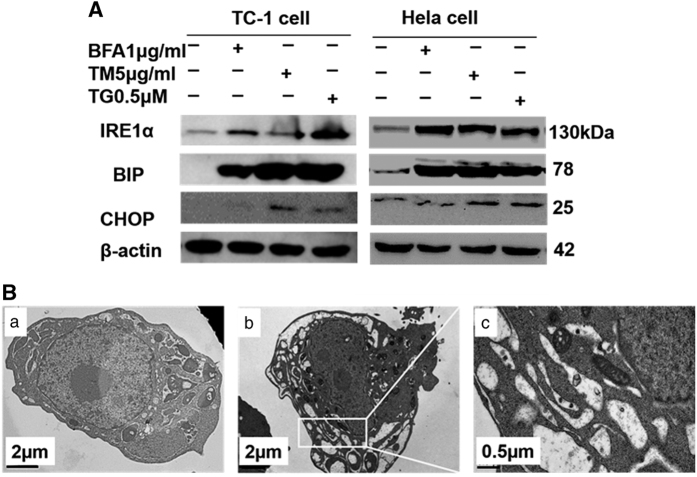
ER stress inducers (BFA, TM, and TG) trigger the UPR. (**A**) Western blot for UPR-related protein levels in TC-1 and HeLa cells treated with BFA, TM, and TG. Western blot analysis of total TC-1 and HeLa cells lysates for UPR-related protein expression, and protein levels were compared with those of *β*-actin. (**B**) Electron microscopic images showing the ultrastructure of BFA-treated TC-1 cell. (a) Representative image of the normal ultrastructure of PBS-treated TC-1 cell. (b) The ultrastructure of BFA-treated TC-1 cell. (c) High magnification image of the ultrastructure of BFA-treated TC-1 cell; solid arrows highlight the swollen endoplasmic reticulum.

The activation of the UPR following ER stress is thought to have a key role in diseases like cancer. We investigated that the UPR activation is a response to ER stress induction in cervical tumor cell lines TC-1 and HeLa. We found that BFA at a concentration of 1 *μ*g/ml, as well as TM (5 *μ*g/ml) and TG (0.5 *μ*M), induced UPR in TC-1 cells and HeLa cells evidenced by increased protein expression of BIP, IRE1a, and C/EBP homologous protein (CHOP), although BFA has little effect on CHOP (Figure 1a). We also observed the swollen ER in the subcellular structure of TC-1 tumor cells by transmission electron microscope (TEM). Some cisterns in swollen ER display a remarkable expansion of the intracisternal space and disappearance of ribosomes from the internal membranes of the cisterns (Figure 1b). This result showed that cervical tumor cells treated by ER stress inducer undergo a remarkable change of activation of UPR.

### BFA significantly promoted TC-1 tumor cell death in a dose-dependent manner

**Figure 2:**
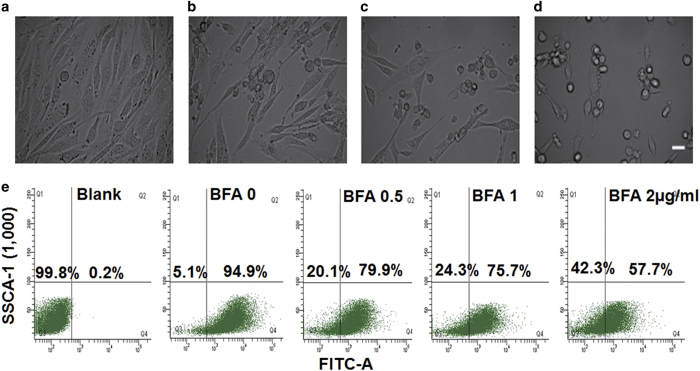
The effect of BFA on TC-1 cancer cells. The morphological changes of the TC-1 cancer cells treated by different concentrations of BFA for 24 h (**a**–**d** represent TC-1 tumor cell that were treated by BFA with 0, 0.5, 1, and 2 *μ*g/ml, respectively). Scale bar: 25 *μ*m. (**e**) Determination of Δψm.TC-1 cells were treated with various concentrations of BFA for 24 h and then stained with rhodamine 123. Loss of Δψm was visualized as a reduction in the fluorescence signal. The proportions of cells with rhodamine 123 staining are shown as the average value of three replicated experiments.

Prolonged ER stress was previously shown to be able to induce cell death *in vitro*.^16^ To determine whether ER stress triggers cell death in our cell model, we observed the morphological changes of tumor cells treated with BFA. After 24 h, BFA treatment of TC-1 tumor cells resulted in the appearance of little black dots at the two poles of the cells, followed by cells becoming more rounded in shape and detaching from the dish (Figures 2a–d). Mitochondrial dysfunction triggers the cell death signaling cascade. Among the sequence of events taking place in mitochondria during the course of cell death, loss of the mitochondria membrane potential (Δψm) appears to be an important event as it is tightly associated with cell death. Rhodamine 123, whose mitochondrial fluorescence intensity decreases quantitatively in response to dissipation of mitochondrial transmembrane potential, was used to evaluate disturbances in Δψm.^17,18^ Flow cytometry analysis revealed that BFA decreased Δψm in a dose-dependent manner, reducing Δψm by 20.1%, 24.3%, or 42.3% following treatment with BFA at concentrations of 0.5, 1, or 2 *μ*g/ml, respectively (Figure 2e). To assess the effects of BFA on TC-1 tumor cell proliferation *in vitro*, we treated the TC-1 tumor cells with increasing concentrations of BFA for 5 days and examined the cell growth by MTT assays. BFA strongly inhibited TC-1 tumor cell proliferation in a dose-dependent manner (Supplementary Figure 1). These results suggested that BFA promotes death and proliferation of the TC-1 tumor cells.

### BFA induced autophagy in TC-1 tumor cells

**Figure 3:**
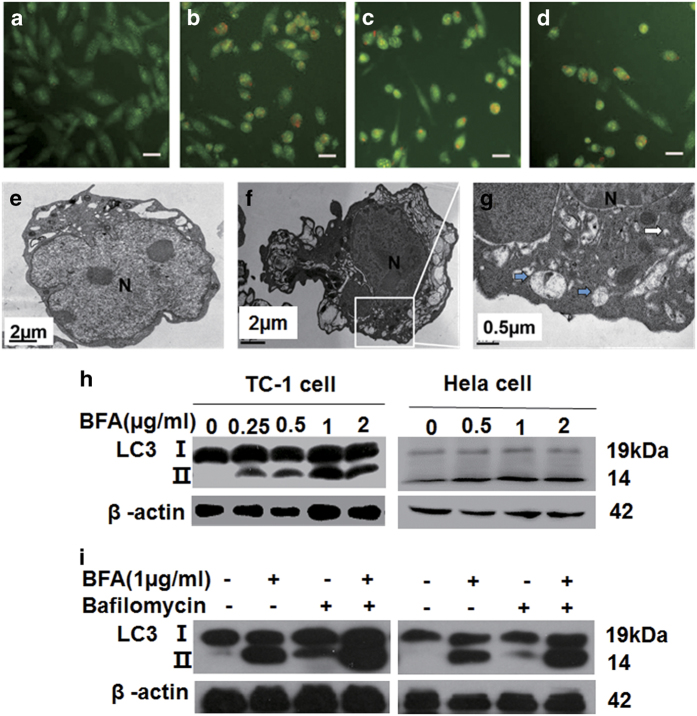
BFA induced autophagy of TC-1 tumor cells. (**a**–**d**) TC-1 tumor cells treated by BFA with 0, 0.5, 1, and 2 *μ*g/ml, respectively, followed by staining with 0.5 *μ*g/ml AO for 30 min at 37 °C. Four random fields were imaged under a fluorescence microscope. Immunoreactive cells with fluorescence were manually counted at ×200 magnification. Scale bar: 25 *μ*m. (**e**) Electron microscopic images showing ultrastructure of a BFA-treated TC-1 cell. TC-1 cells were incubated in PBS or BFA (1 *μ*g/ml) for 6 h and fixed for electron microscopy as described in the text. The normal morphology of cell and intact nucleus of cells in the PBS group. N for the nucleus. Scale bar: 2 *μ*m. (**f**) The ultrastructure of a BFA-treated cell; the cells were distinctly shrinking, as well as the nucleus of the cells. Scale bar: 2 *μ*m. (**g**) A large number of double membrane structures shown as blue solid arrow, which is autophagosome, partly parceled the remnants of organelle, while solid arrows highlight nascent autophagosomes. Scale bar: 0.5 *μ*m. (**h**) Western blot analysis of total TC-1 cell and HeLa cell lysates for LC3, protein levels of LC3II were compared with those of *β*-actin. (**i**) TC-1 cells and HeLa cells were treated with BFA for 24 h in the presence or absence of bafilomycin. Whole-cell lysates were obtained and the content of LC3I and LC3II was determined by western blotting.

ER stress has been reported to induce cell death by concomitant induction of autophagy and apoptosis.^19^ To determine whether BFA increases autophagy in our cell model, we tested autophagy in TC-1 tumor cells treated with BFA using acridine orange (AO) staining (Figures 3a–d). AO interacts with DNA emitting green fluorescence, but when taken up into autolysosomes it becomes protonated forming aggregates that emit bright red fluorescence. BFA treatment significantly increased the amount of red fluorescence detected in TC-1 cells, indicating that autophagy was increased. Autophagy upregulation was also verified using TEM. After exposure to BFA for 24 h, there were a large number of double-membrane autophagic vacuoles presented in BFA-treated cells (Figure 3f), but not in control cells (Figure 3e). Organelles were visible within double-membrane vacuoles at high magnifications (Figure 3g). Western blotting showed that BFA treatment increased LC3II levels in TC-1 tumor cells and HeLa cells in a concentration-dependent manner (Figure 3h). The autophagic flux was also assessed, and bafilomycin decreased the degradation of LC3 in lysosomes, which in turn caused both LC3I and LC3II accumulation in TC-1 tumor cells and HeLa cells (Figure 3i). Collectively, these results showed that BFA can promote autophagy in cervical cancer cells, suggesting that autophagy is the preferred route for degradation of proteins during UPR activation.

### ER stress inducers (BFA, TM, and TG) also triggered apoptosis of TC-1 tumor cells

**Figure 4:**
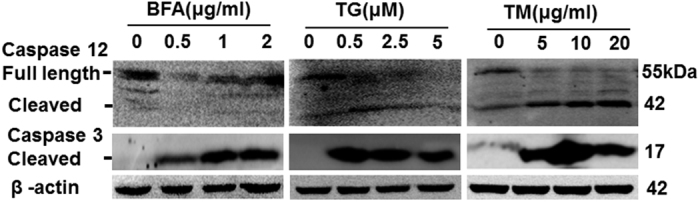
ER stress inducers (BFA, TM, and TG) trigger apoptosis of TC-1 cells. Western blot analysis of caspase-12 and cleaved caspase-3 in cell lysates from TC-1 cells. Protein levels of caspase-12 and cleaved caspase-3 were determined by western blotting compared with those of *β*-actin.

Recent studies reported that ER stress initiates a nonclassical apoptotic pathway, through the cleavage and activation of the caspase-12 downstream of the CHOP.^20,21^ We measured protein levels of caspase-12 and CHOP induced by various ER stressors (including BFA, TM, and TG). Western blotting revealed that ER stressors increased caspase-12 cleavage in a dose-dependent manner evidenced by decreased full-length caspase-12 and increased cleaved caspase-12. Further, we confirmed that ER stress increased the cleaved form of caspase-3, visible as a single band migrating at 17 kDa (Figure 4). However, a role of BFA in the activation of CHOP was very little in TC-1 tumor cells and Atg5+/+ and Atg5−/− MEF cells (Supplementary Figure 2). The results indicated that ER stressors initiate apoptosis of TC-1 tumor cells through the activation of the caspase-12/caspase-3 pathway.

### BFA induced NF-*κ*B activation in TC-1 tumor cells and HeLa cells

**Figure 5:**
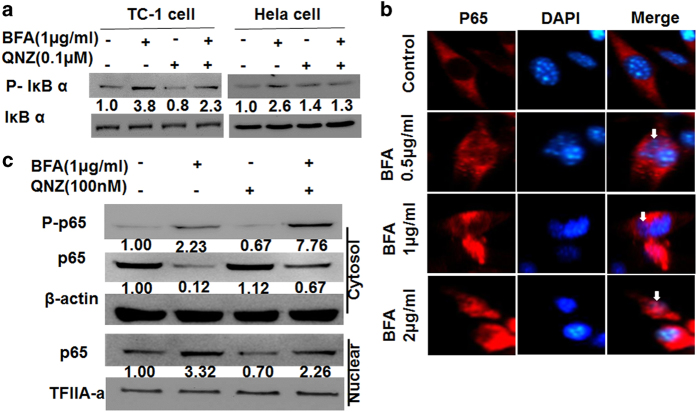
BFA triggers NF-*κ*B pathway activity in TC-1 tumor cells or HeLa cells. (**a**) Western blot analysis of p-IðBa and IðBa in whole-cell lysates from TC-1 cells and HeLa cells, which were treated with BFA for 24 h in the presence or absence of QNZ. (**b**) Immunofluorescence images of TC-1 tumor cells treated with BFA. Arrows point to nuclear transduction of P65. Scale bar: 10 μm. (**c**) TC-1 cells treated with BFA for 24 h in the presence or absence of QNZ. Alternatively, lysates were subjected to subcellular fractionation to evaluate the presence of p65 in the cytoplasm versus the nucleus, using *β*-actin and TFIIA-a as control equal loading of cytoplasmic and nuclear fractions, respectively. Protein levels were determined by western blotting compared with those of *β*-actin.

NF-*κ*B is a transcription factor that mediates antiapoptotic signals in several cancer cell types, and the inhibition of the NF-*κ*B signaling pathway induces apoptosis in cancer cells.^22,23^ NF-*κ*B can block PAR-4-mediated apoptosis by the downregulation of the tracking of PAR-4 receptor GRP78 from the ER to the cell surface.^24^ We found that BFA enhanced the phosphorylation of I*κ*B*α* protein in TC-1 and HeLa cells. QNZ, the NF-*κ*B inhibitor, inhibited the phosphorylation of I*κ*B*α* protein induced by BFA (Figure 5a). The p65 subunit (RelA) of NF-*κ*B plays a critical role in inducing target genes of NF-*κ*B. Immunofluorescence staining showed translocation of NF-*κ*B p65 following ER stress (Figure 5b). Western blot analysis confirmed the translocation of NF-*κ*B p65 from the cytosol to the nucleus. Nuclear translocation of NF-*κ*B following BFA treatment was partly blocked by QNZ (Figure 5c). These results suggest that BFA treatment triggers the activation of the NF-*κ*B signaling pathway.

### QNZ inhibited autophagy and apoptosis, partly enhancing survival of cervical cancer cells following BFA treatment

**Figure 6:**
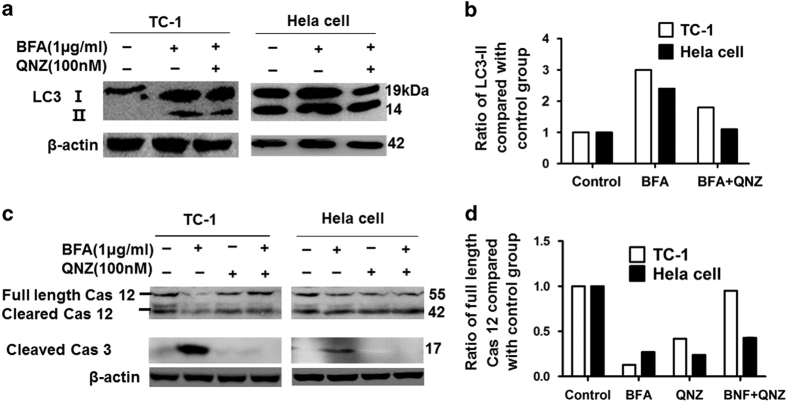
Blocking NF-*κ*B pathway activity by QNZ partly inhibit the autophagy and apoptosis induced by BFA in TC-1 tumor cells or HeLa cells. (**a**) TC-1 and HeLa cells were treated with BFA for 24 h in the presence or absence of QNZ. Whole-cell lysates were obtained and the content of LC3I and LC3II was determined by western blotting. (**b**) Semiquantitative expression of LC3II in western blots was measured by Gray value of LC3II in each group compared with the control group. (**c**) TC-1 cells and HeLa cells were treated with BFA for 24 h in the presence or absence of QNZ; protein levels of caspase-12 and cleaved caspase-3 were determined by western blotting. (**d**) Semiquantitative expression of caspase-12 in western blots was measured by Gray value of LC3II in each group compared with the control group.

**Figure 7:**
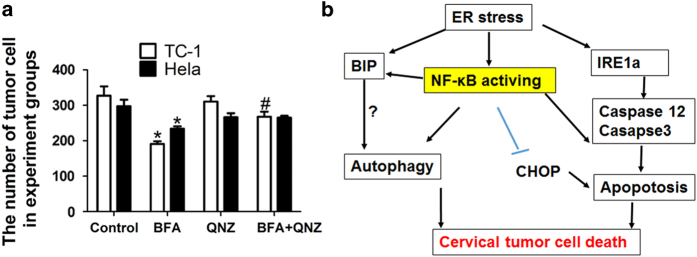
Blocking NF-*κ*B pathway activity by QNZ enhanced the survival of TC-1 cells and HeLa cells under ER stress induced by BFA. (**a**) TC-1 cells and HeLa cells were treated with BFA for 24 h in the presence or absence of QNZ. Cell survival of different groups is calculated by the average of different experiments in triplicate. *P⩽0.05 compared with the control group; ^#^*P*⩽0.05 compared with BFA group. (**b**) Schematic depiction of cross-talk between ER stress, autophagy, apoptosis and NF-*κ*B pathway, this being associated with the fate of cervical cancer cells.

NF-*κ*B is a key transcription factor that orchestrates the expression of many genes associated with inflammation and cancer, which include members of the chemokine/cytokine signaling and cell proliferation and survival pathways. Therefore, we tested whether the NF-*κ*B pathway controls the fate of tumor cells following ER stress induction. We found that blocking the NF-*κ*B pathway with QNZ attenuated the induction of LC3II following treatment with BFA in cervical tumor cells (Figures 6a and b), suggesting that QNZ partly inhibits autophagy-induced BFA. Further, QNZ treatment decreased caspase-12 cleavage as indicated by increasing full-length caspase-12, and abrogated caspase-3 cleavage following BFA in cervical tumor cells (Figures 6c and d). Furthermore, QNZ attenuated the TC-1 tumor cell death induced by BFA (Figure 7a). Interestingly, QNZ enhanced activation of the CHOP pathway in TC-1 tumor cells and Atg5+/+ and Atg5−/− MEF cells (Supplementary Figure 2). These results indicate that blocking NF-*κ*B pathway activity by QNZ inhibited autophagy and apoptosis, partly enhancing survival of cervical cancer cells following BFA treatment.

## Discussion

Our studies showed that induction of ER stress led to the activation of the UPR in cervical tumor cells, which was characterized by elevated levels of IRE1a, GRP-78, and the swelling ER. ER stress significantly promoted cells death by concomitant induction of autophagy and apoptosis in cervical tumor cells by activating the NF-*κ*B pathway. QNZ, a NF-*κ*B pathway inhibitor, decreased the autophagy and apoptosis, and attenuated cervical tumor cell death induced by BFA (Figure 7b). Our study provides evidence that there is cross-talk between ER stress, autophagy, apoptosis, and NF-*κ*B pathway in cervical tumor cells, which controls the fate of the tumor cells by sensing changes in extracellular microenvironment.

In response to diverse stress, the ER initiates an adaptive response called the UPR with an aim to restore ER homeostasis. If the stress signal is severe and/or prolonged, ER stress triggers cell death pathways. The question about what determines the switch between prosurvival and prodeath UPR signals is an area of much interest, and the answer to this question should promote the development of novel drugs targeting the prodeath UPR signals as an anticancer therapeutic strategy.^25^ However, a greater understanding of the integration of the UPR itself with other signaling pathways and how it relates to cell fate control is necessary.

ER stress-induced cell death can be a result of the autophagy pathway.^26,27^ Autophagy induces tumor death by increased digestion of survival factors over death factors, or digestion of cellular necessary components.^28^ Thus, the impact of autophagy on cell survival during ER stress is probably contingent on the status of the cells, which could be explored for tumor-specific therapy. In this report, we show that BFA effectively triggers autophagy and activation of NF-*κ*B signaling. ER stress induced LC3II conversion and autophagosome formation accompanied with elevated IRE1. IRE1 is crucial for autophagosome formation and LC3II conversion after treatment with ER stressors. This result is consistent with a previous report, which suggested that IRE1, rather than PERK, links UPR to autophagy.^29^ Alternatively, some studies showed ER stress-induced autophagy via PERK/eIF2*α* phosphorylation.^30^ ER stress-induced autophagy may be mediated by different mechanisms in different cell models. By virtue of phosphorylation of I*κ*B, which lead to the translocation of NF-*κ*B p65, ER stressors enhance NF-*κ*B activation in cervical cancer cells, and inhibition of the NF-*κ*B pathway prevented BFA-induced autophagy. The results reveal that blocking NF-*κ*B signaling could inhibit autophagic cell death induced by ER stress.

ER stress-induced cell death could also be a result of the apoptosis pathway.^31,32^ Environmental factors contribute to the activation of ER stress, and as a result, cancerous cells must possess ways to adapt and prevent the fate of ER stress-induced apoptosis. Recent studies show that caspase-12 specifically participates in the apoptotic signaling induced by ER stress.^33,34^ Similarly, ER stressors initiated apoptosis of TC-1 tumor cells through activation of caspase-12. QNZ treatment decreased caspase-12 cleavage as indicated by increasing full-length caspase-12, and abrogated caspase-3 cleavage following BFA in cervical tumor cells, without blocking the inhibition of caspase-12 and caspase-3 mRNA following BFA treatment (Supplementary Figure 3). Caspase-12 and caspase-3 are activated in the apoptotic cell both by extrinsic (death ligand) and intrinsic (mitochondrial) pathways. Inhibitor of apoptosis (IAP) directly regulates apoptosis by preventing the activation of caspase-3.^35^ It is possible that QNZ inhibits the activation of caspase-12 or caspase-3 in cells under ER stress by enhancing the expression of IAP family members.

Interestingly, QNZ simultaneously enhanced protein expression of CHOP, another proapoptotic gene downstream of the ER stress pathway, in TC-1 tumor cells after treatment with BFA. Blocking the NF-*κ*B pathway using QNZ resulted in ER stress initiating apoptosis through activation of the CHOP pathway rather than with activation of the caspase-12/caspase-3 pathway. The results of the current study provide evidence that CHOP links ER stress to NF-*κ*B activation, which is consistent with previous studies.^36,37^

Cervical carcinoma is a growing menace to women’s health worldwide, and is one of the leading causes of death in women worldwide. Although HPV is considered to be the major cause of cervical cancer, yet the viral infection alone is not sufficient for cancer progression. Activating the NF-*κ*B signaling pathway promotes proliferation, invasion and metastasis of cervical cancer cells, thus NF-*κ*B pathway inhibitors are being suggested as good anticancer agents in cervix carcinoma.^11,12^ However, based on all results, it appears that inhibition of NF-*κ*B activation may not be a safe strategy in the development of novel agents to treat cervical cancer.^13,14^

In conclusion, our results indicate that there is a cross-talk between ER stress, autophagy, apoptosis and NF-*κ*B pathway, which helps determine the fate of cervical cancer cells. Careful evaluation should be given to the use of NF-*κ*B pathway inhibitors to treat cervical cancer in combination with drugs that induce tumor cell death through ER stress induction.

## Materials and methods

### Chemicals and reagents

BFA,TM, TG, and QNZ were purchased from Sigma-Aldrich (St. Louis, MO, USA), were diluted in dimethyl sulfoxide, and stored at −20 °C. Rabbit anti-LC3 antibody (cat. no. L7543), 4′,6′-diamidino-2-phenylindole (DAPI), and rhodamine 123 were all purchased from Sigma-Aldrich. Antibodies for NF-*κ*B p65 (cat. no. 8284), IкB*α* (cat. no. 4812), p-IкB*α* (cat. no. 5209), P-IKK*α*/*β* (cat. no. 9958), BIP (cat. no. 3177), IRE1*α* (cat. no. 3294), CHOP (cat no. 2895), caspase-12 (cat no. 2202), and cleaved-caspase-3 (cat. no. 9654), all were purchased from Cell Signaling Technology (Danvers, MA, USA); anti-TFIIAa and secondary antibodies were from Santa Cruz Biotechnology (Dallas, TX, USA).

### Cell culture and treatment

Two cervical cancer cell lines (TC-1 tumor cells and HeLa cells) were used in this study. They were cultured in RPMI-1640 medium (Invitrogen, San Diego, CA, USA). Atg5+/+ and Atg5−/− mouse embryonic fibroblast (MEF) cells were maintained in DMEM (Invitrogen). Both media were supplemented with 10% (v/v) fetal bovine serum and 1% (v/v) penicillin/streptomycin. All cells were maintained in a 37 °C, 95% humidity, and 5% carbon dioxide environment. For experimental purposes, the cells were grown in serum-free RPMI-1640 medium before and during treatment. For the test of autophagic flux, cells were exposed to 100 nM BFA. For inhibition of the NF-*κ*B pathway, cells were incubated with 100 nM QNZ (Sigma-Aldrich) for 1 h before BFA treatment.

### Transmission electron microscope

Cells were fixed in 2.5% glutaraldehyde in 0.1 M cacodylate buffer, pH 7.4, postfixed in 1% osmium tetroxide, pH 7.2, and then treated with 0.5% tannic acid, 1% sodium sulfate, cleared in 2-hydroxypropyl methacrylate. Cells were next embedded in Ultracut (Leica, Wetzlar, Germany) and sliced into 60-nm sections. Ultrathin sections were stained with uranyl acetate and lead citrate, and examined with a JEM-1230 TEM (JEOL, Tokyo, Japan).

### Determination of Δψ_m_

Rhodamine 123 was used to evaluate changes in Δψ_m_. Cells (1×10^5^) were placed in 6-well plates and treated with BFA at the concentrations indicated for 24 h. The cells were then collected and resuspended in 1 ml PBS containing 10 *μ*g/ml rhodamine 123 for 15 min at 37 °C, and then analyzed using the FACS Vantage flow cytometer (Beckman Counter-Epics XL; Beckman Coulter Inc. SA, Nyon, Switzerland). Results were expressed as the proportion of cells exhibiting low mitochondrial membrane potential estimated by the reduced rhodamine 123 uptake.

### AO staining

AO is used in autophagy assays and stains autolysosomes.^38^ Briefly, cells were treated with indicated concentrations of BFA (0, 0.5, 1, and 2 *μ*g/ml, respectively), followed by staining with 0.5 *μ*g/ml AO (Sigma-Aldrich) for 30 min at 37 °C and then washed once with PBS. The coverslips were mounted onto glass slides with glycerin and analyzed on an Olympus FV1000 fluorescence microscope (Olympus, Tokyo, Japan).

### MTT assay and cell viability assays

The MTT assay was performed as described previously.^39^ In brief, the cells were cultured in phenol red-free medium in 24-well plates. Cytotoxicity of BFA was determined using an MTT Cell Viability Assay Kit from ATCC Bioproducts (Manassas, VA, USA) following the manufacturer’s instructions. The 96-well microplates were read using a Spectra Max M5 Microplate Reader (Molecular Devices, Sunnyvale, CA, USA), and absorbance was measured at 570 nm. Cell viability assays were measured by trypan blue exclusion assay. Each data point was the average of three different experiments in duplicates.

### Light and immunofluorescence microscopy

Cells were processed for immunofluorescence staining according to established protocols.^40^ Briefly, cells (2×10^4^) were plated on 24-well plates and treated with BFA for 8 h. Then, cells were fixed with 4% PFA in PBS for 15 min at RT. After washing three times with PBST, the cells were blocked in PBS with 5% BSA and 0.05% Triton X-100 for 30 min at RT. The cells were washed and incubated with anti-p65 overnight at 4 °C. Subsequently, the cells were washed again and then incubated with secondary antibodies for 1 h. After washing three times, the cells were stained with Alexa Fluor 555 goat anti-rabbit IgG (cat. no.1683674) from Life Technologies (Waltham, MA, USA) for 30 min. The cells were stained with DAPI (5 *μ*g/ml; Sigma-Aldrich) for 5 min and then washed with PBS. The coverslips were mounted onto glass slides with glycerin and analyzed on an Olympus FV1000 microscope.

### Western blot analysis

Cells were lysed in RIPA buffer (Pierce, Rockford, IL, USA) supplemented with protease inhibitor cocktail and phosphatase inhibitors (Sigma). Total cellular extracts (50 *μ*g) were separated in 4–20% SDS-PAGE precast gels (Bio-Rad Laboratories, Berkeley, CA, USA) and transferred onto nitrocellulose membranes (Millipore Corp., Bedford, MA, USA). Membranes were first probed with BIP (1 : 1000), IRE1*α* (1 : 1000), CHOP (1 : 1000), LC3 (1 : 1000), caspase-12 (1 : 1000), cleaved caspase-3 (1 : 500), P-I*κ*B*α* (1 : 1000), I*κ*B*α* (1 : 1000), p65 (1 : 1000), TFIIA-a, and *β*-actin (1 : 1000) antibodies, followed by goat anti-rabbit secondary antibody conjugated with HRP (1 : 5000; Millipore). Protein detection was performed using ECL Kit (Bio-Rad Laboratories; cat. no. PK207480). The data were adjusted to actin expression to eliminate the variations. For stripping, membranes were submerged for 30 min at 55–60 °C in a buffer containing 100 mM 2-mercaptoethanol, 2% (v/v) SDS and 62.5 mM Tris-HCl, pH 6.7, with agitation, and then were washed three times with PBST.

### Statistical analysis

All statistical analysis was performed using the GraphPad Prism Software 6.0 (GraphPad Software Inc., San Diego, CA, USA). The data were presented as the mean±S.E.M. When applicable, unpaired Student’s *t*-test or one-way ANOVA, followed by Tukey’s multiple comparison test were used to determine significance. *P*<0.05 was considered to be statistically significant.

## Additional Information

**Publisher’s note** Springer Nature remains neutral with regard to jurisdictional claims in published maps and institutional affiliations.
