# Supplementary figures and images for: NF-κB pathway link with ER stress-induced autophagy and apoptosis in cervical tumor cells
Source: Cell Death Discov. 2017 Sep 11;3:17059–. doi: 10.1038/cddiscovery.2017.59 (PMC5592653; doi:10.1038/cddiscovery.2017.59)

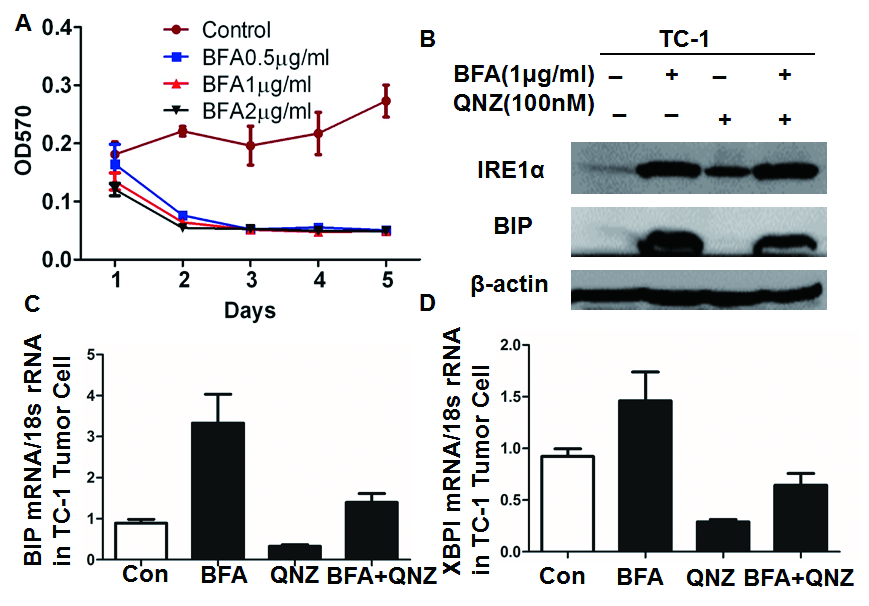

Supplement: Supplementary Figure 1 [file cddiscovery201759-s1.tiff]

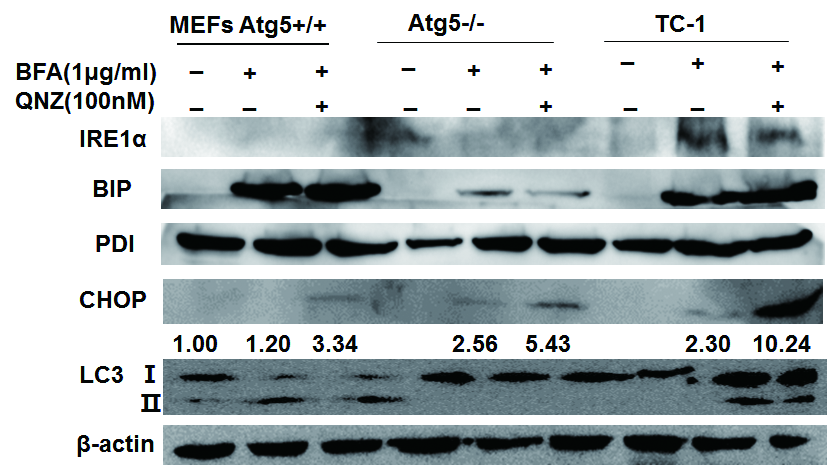

Supplement: Supplementary Figure 2 [file cddiscovery201759-s2.tiff]

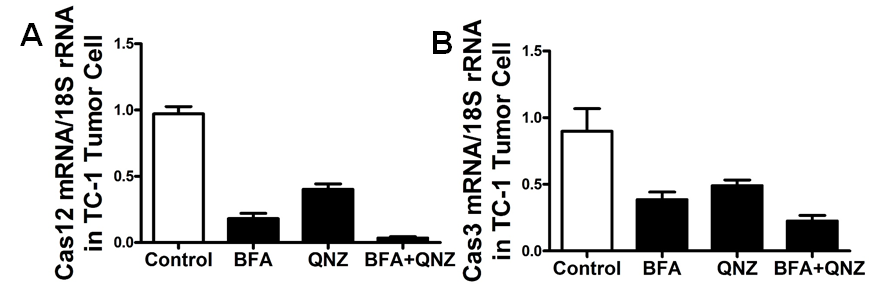

Supplement: Supplementary Figure 3 [file cddiscovery201759-s3.tiff]
